# Patterns of antenatal corticosteroid administration in a cohort of women with diabetes in pregnancy

**DOI:** 10.1371/journal.pone.0229014

**Published:** 2020-02-27

**Authors:** Jeremy F. Tuohy, Frank H. Bloomfield, Jane E. Harding, Caroline A. Crowther

**Affiliations:** Liggins Institute, University of Auckland, Auckland, New Zealand; Centre Hospitalier Universitaire Vaudois, FRANCE

## Abstract

Antenatal corticosteroids administered to the mother prior to birth decrease the risk of mortality and major morbidity in infants born at less than 35 weeks’ gestation. However, the evidence relating to women with diabetes in pregnancy is limited. Clinical guidelines for antenatal corticosteroid administration recommend that women with diabetes in pregnancy are treated in the same way as women without diabetes, but there are no recent descriptions of whether contemporary practice complies with this guidance. This study is a retrospective review of antenatal corticosteroid administration at a New Zealand tertiary hospital in women with diabetes in pregnancy. We found that in this cohort, for both an initial course at less than 35 weeks’ gestation and repeat courses at less than 33 weeks’, the administration of antenatal corticosteroid to women with diabetes in pregnancy is largely consistent with current Australian and New Zealand recommendations. However, almost 25% of women received their last dose of antenatal corticosteroid at or beyond the latest recommended gestation of 35 weeks’ gestation. Pre-existing diabetes and planned caesarean section were independently associated with an increased rate of antenatal corticosteroid administration. We conclude that diabetes in pregnancy does not appear to be a deterrent to antenatal corticosteroid administration. The high rates of administration at gestations beyond recommendations, despite the lack of evidence of benefit in this group of women, highlights the need for further research into the risks and benefits of antenatal corticosteroid administration to women with diabetes in pregnancy, particularly in the late preterm and early term periods.

## Introduction

There is high-quality evidence demonstrating that antenatal corticosteroids (ANC) administered to the mother prior to birth decrease the risk of mortality and major morbidity in infants born at less than 35 weeks’ gestation [1]. However, the evidence for benefit is limited in particular subgroups of women, such as those with diabetes in pregnancy (DIP) [1,2]. The Australian and New Zealand guidelines for the clinical use of ANC [3] recommend that, in the absence of trials of ANC administration conducted in women with DIP, ANC should be administered to women with DIP in the same way as to women without diabetes.

Women with pre-existing diabetes [4] and gestational diabetes [4,5] are more likely than women without diabetes to receive ANC, but it is not clear if this is due to the increased rate of preterm birth in women with DIP [6,7] or because their infants are more likely to develop complications of preterm birth [8], or both.

ANC therapy causes hyperglycaemia and in women with DIP this may require an increase in therapy to maintain euglycaemia [9–11]. The Australian and New Zealand ANC guidelines advise that the blood glucose concentrations of women with DIP should be monitored closely after ANC administration [3]. Severe hyperglycaemia can result in diabetic ketoacidosis which has been reported to be associated with a risk of stillbirth in up to 30% of cases [12]. Maternal hyperglycaemia in labour is associated with neonatal hypoglycaemia [13,14]. Both of these complications of ANC administration may impact upon decision-making around administration of ANC to women with DIP.

Infants of women with DIP are more likely to suffer from respiratory distress syndrome, even at late-preterm gestations [15]. The Australian and New Zealand ANC guidelines do not recommend the use of ANC after 35 weeks’ gestation or prior to elective caesarean section at term unless there is evidence of fetal lung immaturity [3]. However, administration of ANC in the late preterm period has increased over the last 20 years [5,16] and many practitioners in Australia and New Zealand report prescribing ANC in the late preterm and early term period prior to caesarean section [17]. Some professional bodies elsewhere recommend this practice [18] while others state that ANC administration could be considered [19], particularly in situations where infants are at greater risk of respiratory complications [20].

An initial course of ANC improves infant outcomes when given within 7 days of birth [1]. If a woman does not give birth during this time period but is less than 33 weeks’ pregnant and remains at risk of preterm birth, the Australian and New Zealand Guidelines recommend up to three subsequent courses of a single dose of ANC [3]. No adverse outcomes up to mid-childhood have been reported following repeat courses of ANC compared to a single course [21]. Despite the demonstrated benefit of repeat courses of ANC, ongoing concern regarding potential adverse effects is reflected in the declining rates of repeat courses of ANC reported over the last 5 years [5,22,23].

Although DIP is a common medical complication in pregnancy, affecting 9% of women in New Zealand [24], there are few data regarding current practice for ANC administration in these women, who comprise a small percentage of all women who receive ANC, and types of diabetes are seldom differentiated [5,25–27].

## Aims

The aims of the study were to describe the patterns of ANC administration in a cohort of women with DIP and to determine in this cohort: the proportion of women who received ANC; maternal factors associated with women receiving ANC; the proportion of women receiving ANC in accordance with the local guidelines [3], and the influence of mode of birth on the proportion of women receiving ANC.

## Materials and methods

### Study design

This is a retrospective study in a New Zealand tertiary hospital with a dedicated antenatal clinic for women with DIP. All women with a diagnosis of DIP giving birth after 22 weeks’ gestation between 2006 and 2016 were identified from the hospital database. The hospital database was used to extract maternal demographic data (ethnicity, maternal age, parity, year of birth, body mass index (BMI)), pregnancy data (multiple pregnancy, mode of birth, type of diabetes) and infant data (gestation at birth, birthweight and centile, sex, admission to neonatal unit). The mode of birth was categorised as spontaneous vaginal birth, operative vaginal birth and caesarean section. Caesarean section was described as elective caesarean section and in established labour or not, and emergency caesarean section and in established labour or not. Drug charts for each woman were reviewed to identify any ANC administration for the purpose of fetal lung maturation (corticosteroid drug, dose, schedule, number of doses, date and time of administration). Women with a multiple pregnancy and women who gave birth to more than one child over the study period were included. Women who had a stillborn infant were excluded.

The data from the hospital database were downloaded into an anonymised secure research database and the ANC data added. An independent second data extraction of 10% of records identified an error rate in the research database of less than 1%.

### Statistical analysis

The cohort is described using numbers and percentages. Multivariate regression models were used to compare the rate of ANC administration for the predefined maternal variables (ethnicity, birth year, maternal age, parity, body mass index, multiple pregnancy, type of diabetes, mode of birth and gestational age at birth). P-values less than 0.05 were considered statistically significant. Data are presented as number, percent and relative-risks with 95% confidence intervals where appropriate.

The study was approved by the Northern B Health and Disability Ethics Committee (16NTB216). The ethics committee approved that access to potentially identifiable data for this research without written consent was acceptable.

## Results

A total of 7317 women with a pregnancy complicated by DIP were identified. Thirty-five women were excluded, 30 due to antenatally diagnosed stillbirth and five who gave birth to live-born infants before 24 weeks’ gestation, since there were insufficient women in this group for analysis, and the policy of the hospital over the period of this study was not to routinely administer corticosteroids to women at risk of giving birth before 24 weeks [28]. Of the remaining 7282 women with DIP, an initial course of ANC was administered to 8.9% (647/7282) and a repeat course to 1.5% (113/7282).

Overall, 1432 doses of ANC were administered to 647 women. Betamethasone 11.4 mg was used for 97% of these doses (1389/1432), 27 doses were administered as part of a randomised trial comparing dexamethasone and betamethasone (2%), 7 doses (<1%) were dexamethasone and in 9 (1%) the drug used was not recorded. Of the 647 women who received ANC, 90% (586/647) received a complete initial course of two doses, almost all at 24-hour intervals (94%, 551/584). Of the 113 women receiving a repeat course of ANC, 90% (101/113) received a single dose for each repeat course. Eight women (8.0% of those receiving a repeat course and 1.2% of all women receiving ANC) received more than the recommended number of 3 repeat courses, each of a single dose. Fifty-eight percent (375/647) of women gave birth within 7 days of the ANC administration, 81% (304/375) after the first course and 19% (71/375) after a repeat course of ANC.

### Maternal and neonatal factors influencing rates of ANC administration

Receipt of ANC was not related to ethnicity, age, year of birth, parity or BMI (Table 1). Women were more likely to receive ANC if they had type 1 diabetes rather than gestational diabetes, gave birth preterm, gave birth by caesarean section or had a multiple pregnancy.

**Table 1:** Receipt of ANC in women with DIP and different demographic and obstetric characteristics.

Characteristic	All women with DIP		Women with DIP receiving ANC		OR (95%CI)		P value^a^
	n	%	n	Rate/100			
**Ethnicity**							
*NZ European*	*1378*	*19*.*0*	*170*	*12*.*4*	*Reference*		*-*
Asian	2148	29.5	126	5.9	0.76	(0.53–1.08)	0.13
Māori	614	8.4	73	11.9	0.88	(0.69–2.04)	0.54
Pacifica	1249	17.2	123	10.0	0.88	(0.59–1.31)	0.53
Indian	1165	16.0	104	8.9	0.91	(0.62–1.33)	0.63
Other	728	10.0	51	7.0	0.74	(0.59–1.31)	0.18
**Birth Year**							
*2006–2009*	*1914*	*26*.*3*	*199*	*10*.*4*	*Reference*		*-*
2010–2013	3014	35.5	276	9.2	0.95	(0.63–1.62)	0.73
2014–2016	2354	38.2	172	7.3	0.80	(0.59–1.07)	0.13
**Maternal Age**							
*25–35*	*4091*	*56*.*2*	*318*	*7*.*8*	*Reference*		*-*
<25	482	6.6	41	8.5	1.09	(0.67–1.77)	0.74
>35	2709	37.2	288	10.6	1.13	(0.89–1.45)	0.31
**Parity**							
*0*	*3077*	*42*.*3*	*242*	*7*.*9*	*Reference*		*-*
1	2408	33.1	215	9.0	1.11	(0.75–1.46)	0.47
>1	1797	24.7	190	10.6	1.05	(0.75–1.45)	0.79
**BMI**							
*20–24*	*2185*	*30*.*6*	*168*	*7*.*7*	*Reference*		-
*<20*	497	7.0	24	4.8	0.95	(0.53–1.70)	0.87
*25–29*	1794	25.1	157	8.8	1.22	(0.90–1.67)	0.20
*>30*	2666	37.3	278	10.4	1.12	(0.81–1.54)	0.50
**Multiple Pregnancy**							
*Singleton*	*7155*	*98*.*3*	*581*	*8*.*1*	*Reference*		*-*
Multiple	127	1.7	66	52.0	1.95	(1.13–3.38)	0.02*
**Type of Diabetes**							
*GDM*	*6048*	*83*.*1*	*443*	*7*.*3*	*Reference*		*-*
Type 1	381	5.2	80	21.0	1.92	(1.29–2.87)	0.001*
Type 2	853	11.7	124	14.5	1.30	(0.93–1.81)	0.12
**Mode of Birth**							
*SVB*	*3542*	*48*.*6*	*160*	*4*.*5*	*Reference*		-
OVB	793	10.9	36	4.5	1.39	(0.88–2.22)	0.15
CS Emergency	1514	20.8	224	14.8	2.13	(1.57–2.90)	<0.0001**
CS Elective	1433	19.7	227	15.9	4.83	(3.60–6.48)	<0.0001**
**Gestation at Birth (weeks)**							
*37 or more*	*6322*	*86*.*8*	*186*	*3*.*0*	*Reference*		*-*
32 or less	169	2.2	163	97.0	871	(347–2026)	<0.0001**
33–34	189	2.6	141	74.6	99	(67–147)	<0.0001**
35–36	602	8.3	157	26.1	10.1	(7.8–13.0)	<0.0001**

GDM Gestational Diabetes, CS caesarean section, SVB spontaneous vaginal birth, OVB operative vaginal birth.

^a^ *p* values calculated using multiple logistic regression analyses.

* Denotes significance at p<0.05

** Denotes significance at p<0.0001

**Fig 1:**
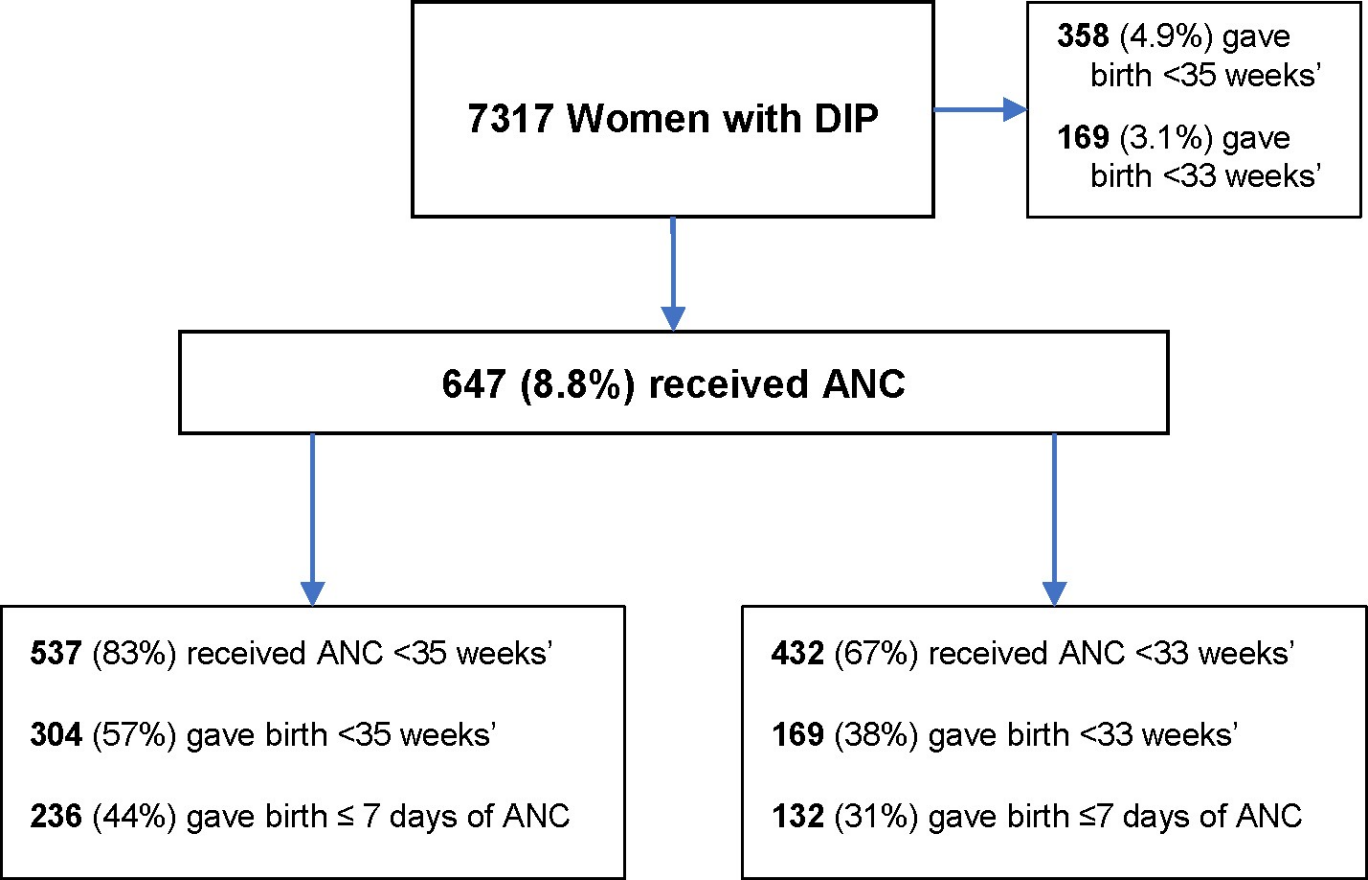
The relationship between the gestational age at ANC administration and birth. ANC–Antenatal corticosteroids, DIP–Diabetes in pregnancy.

Of all women receiving ANC, 83% (537/647) received their initial dose before 35 weeks’ gestation; of these women 57% (304/537) birthed at less than 35 weeks’ gestation and 44% (236/537) birthed within 7 days of the ANC administration. Of all women giving birth before 35 weeks’, 85% (304/358) received ANC and 64% (227/358) received ANC within 7 days of birth (Fig 1).

Of all women receiving ANC, 67% (432/647) received their initial dose before 33 weeks’ gestation; of these women 38% (169/432) gave birth at less than 33 weeks’ gestation and 31% (132/432) birthed within 7 days of ANC administration. Of all women giving birth before 33 weeks’ gestation, 96% (163/169) received ANC and 81% (132/169) received either an initial (n = 86) or repeat (n = 46) course of ANC within 7 days of birth (Fig 1). Of all women receiving ANC, 23% (147/647) received their last dose of ANC at 35 weeks’ or more and 8% (53/647) at 37 weeks or more.

### Administration of ANC prior to caesarean section

Of the total cohort, 40.5% (2947/7282) of women gave birth by caesarean section, of whom 18% (452/2947) received ANC, 8.0% (236/2947) within 7 days of birth. For women receiving ANC, 70% (452/647) gave birth by caesarean section and 60% (388/647) gave birth by elective caesarean section or by emergency caesarean section without labour (Table 2). Emergency caesarean section, elective caesarean section and caesarean section not in established labour, but not caesarean section in established labour, were independently associated with a greater odds of ANC administration compared to a spontaneous vaginal birth (Table 2).

**Table 2:** Comparison of rates of ANC administration by mode of birth.

Characteristic	All Women with DIP		Women with DIP receiving ANC		OR (95%CI)		P Value^a^
	n	%	n	Rate/100			
**Total**	7282		648				
**Vaginal Birth**							
*SVB*	*3542*	*48*.*6*	*160*	*4*.*5*	*Reference*		
OVB	793	10.9	36	4.5	1.39	(0.88–2.21)	0.16
**Caesarean Section**							
**-Type**							
CS Emergency	1514	20.8	224	14.8	2.13	(1.57–2.92)	<0.0001
CS Elective	1433	19.7	228	15.9	4.83	(3.60–6.48)	<0.0001
**-Timing**							
CS IEL	884	12.1%	71	8.0	1.42	(0.95–2.12)	0.09
CS NIEL	2063	28.3%	381	18.5	4.5	(3.45–5.92)	<0.0001

SVB–Spontaneous Vaginal Birth, OVB–Operative Vaginal Birth, CS–Caesarean Section, IEL–In Established Labour, NIEL–Not in Established Labour.

^a^ p values calculated using multiple logistic regression analyses.

For women giving birth between 35 and 38 weeks’ the rate of ANC administration varied by different modes of birth as follows: elective caesarean section 11.2%; emergency caesarean section 6.5%; operative vaginal birth 3.1%, and spontaneous vaginal birth 2.3%.

## Discussion

This study describes the patterns of administration of ANC in a New Zealand cohort of women with DIP. Almost all women with DIP giving birth before 35 weeks’ gestation received at least one course of ANC and more than one half of women who received ANC received them within 7 days of birth. These rates are consistent with or greater than those reported for cohorts of women from the general population by the Australian and New Zealand Neonatal Network [29], the Canadian Perinatal Network [30] and other large tertiary level units [5,27].

In our cohort, women with pre-existing diabetes, who are known to have poorer glycaemic control than women with gestational diabetes [31], were more likely to receive ANC. This suggests that DIP and the need for blood glucose monitoring with potential increase in therapy required to maintain euglycaemia, does not deter obstetricians from prescribing ANC to women with DIP. This is consistent with a recent survey of practitioner ANC prescribing practice [17] and the advice from the current guidelines [17].

The rate of administration of a complete course of ANC (90%) was higher than rates reported for the general population in Australia and New Zealand [29]. Since rates of completion of a course of ANC are higher in women giving birth electively as opposed to those in preterm labour [4], the high rate of birth by caesarean section in this cohort, particularly caesarean section not in established labour, likely contributed to the high rate of ANC course completion.

There is a greater opportunity to administer ANC to women giving birth electively and infants born without labour are at greater risk of respiratory morbidity than infants born after a labour [32]. Both of these factors likely contribute to the high rate of ANC prior to elective birth. Although there is no proven survival benefit for administration of ANC to infants born at 35 weeks’ or more, there is some evidence that ANC administration in the late preterm period decreases respiratory complications in the neonate and decreases the rate of neonatal unit admission [1,33]. However, the reported association between ANC administration in the late preterm period and neonatal hypoglycaemia [14,33,34] may pose additional risks to infants of women with DIP who have been reported to have a 50% risk of developing neonatal hypoglycaemia [35].

Elective caesarean section at less than 39 weeks’ gestation is associated with an increase in the rate of neonatal respiratory complications [36,37] and infants born to mothers with DIP are at increased risk of respiratory morbidity compared to those born to mothers without DIP [8]. A systematic review of ANC administration prior to elective caesarean section at term reported a decrease in the risk of respiratory distress syndrome, transient tachypnoea of the newborn, admission to the neonatal intensive care unit for respiratory morbidity compared to placebo [38]. There are no reliable data to assess the rate of respiratory morbidity in infants of mothers with DIP exposed to ANC.

The current Australian and New Zealand guidelines for ANC administration [3] recommend that an elective caesarean section is delayed until 39 weeks’ gestation or more if possible, in order both to obviate the requirement for ANC administration and to reduce respiratory complications in the newborn. As only 17% of women in our cohort underwent elective caesarean section at 39 weeks’ or more, greater emphasis on adherence to this recommendation may result in a significant reduction in neonatal respiratory morbidity and neonatal unit admission without the requirement for ANC therapy. Alternatively, if it is considered necessary for a women with DIP to give birth by elective caesarean section at less than 39 weeks’, the current Australian and New Zealand guidelines [3] are to administer ANC if there is evidence of lung immaturity. Unfortunately, there is no evidence specifically in women with DIP comparing the relative risks and benefits of either delaying birth by elective caesarean section until 39 weeks or administering ANC prior to 39 weeks.

Repeat courses of ANC administered more than 7 days after an initial course decrease the rate of serious infant outcomes including respiratory disease [39]. The guidelines [3] recommend the use of repeat courses of ANC in women who are less than 33 weeks’ gestation, at risk of preterm birth within the next 7 days and who have not received ANC within the last 7 to 14 days. In our cohort, most women (81%) giving birth at less than 33 weeks received ANC within 7 days of birth, and very few (1.2% of women receiving ANC) received more than the recommended number of repeat courses of ANC. This indicates that a strategy of utilizing a limited number of repeat courses of ANC to women giving birth at less than 33 weeks’ gestation can result in the administration of ANC within 7 days of birth to most women without exceeding the recommend number of doses.

The main limitation of this study is its retrospective design, and practice may have changed since the inception of the cohort in 2006. The strength of this study is that it is the largest reported cohort of women with DIP which identified all women who received ANC, allowing analysis of the patterns of ANC administration according to both gestation at administration and gestation at birth.

We conclude that administration of ANC to women with DIP in this cohort was largely consistent with current recommendations for both an initial course at less than 35 weeks’ and repeat courses at less than 33 weeks’, indicating that diabetes in pregnancy does not appear to be a deterrent to ANC administration at these gestations. However, almost 25% of all women received their last dose of ANC at or beyond the latest recommended gestation despite the lack of evidence of benefit in this group. This appears to be due, at least in part, to administration of ANC in women with DIP birthing via planned caesarean section, and in women with pre-existing diabetes. Further research is required into the risks and benefits of ANC administration to women with DIP, particularly those giving birth in the late preterm and early term periods.
